# Intraperitoneal Infection of Wild-Type Mice with Synthetically Generated Mammalian Prion

**DOI:** 10.1371/journal.ppat.1004958

**Published:** 2015-07-02

**Authors:** Xinhe Wang, Gillian McGovern, Yi Zhang, Fei Wang, Liang Zha, Martin Jeffrey, Jiyan Ma

**Affiliations:** 1 Center for Neurodegenerative Science, Van Andel Research Institute, Grand Rapids, Michigan, United States of America; 2 Department of Molecular and Cellular Biochemistry, Ohio State University, Columbus, Ohio, United States of America; 3 Animal and Plant Health Agency, Lasswade Laboratory, Pentlands Science Park, Penicuik, Midlothian, Scotland; 4 Key Laboratory of Brain Functional Genomics (East China Normal University), Ministry of Education, Shanghai Key Laboratory of Brain Functional Genomics (East China Normal University), School of Life Sciences, East China Normal University, Shanghai, China; Istituto Superiore di Sanità, ITALY

## Abstract

The prion hypothesis postulates that the infectious agent in transmissible spongiform encephalopathies (TSEs) is an unorthodox protein conformation based agent. Recent successes in generating mammalian prions *in vitro* with bacterially expressed recombinant prion protein provide strong support for the hypothesis. However, whether the pathogenic properties of synthetically generated prion (rec-Prion) recapitulate those of naturally occurring prions remains unresolved. Using end-point titration assay, we showed that the *in vitro* prepared rec-Prions have infectious titers of around 10^4^ LD_50_ / μg. In addition, intraperitoneal (i.p.) inoculation of wild-type mice with rec-Prion caused prion disease with an average survival time of 210 – 220 days post inoculation. Detailed pathological analyses revealed that the nature of rec-Prion induced lesions, including spongiform change, disease specific prion protein accumulation (PrP-d) and the PrP-d dissemination amongst lymphoid and peripheral nervous system tissues, the route and mechanisms of neuroinvasion were all typical of classical rodent prions. Our results revealed that, similar to naturally occurring prions, the rec-Prion has a titratable infectivity and is capable of causing prion disease via routes other than direct intra-cerebral challenge. More importantly, our results established that the rec-Prion caused disease is pathogenically and pathologically identical to naturally occurring contagious TSEs, supporting the concept that a conformationally altered protein agent is responsible for the infectivity in TSEs.

## Author Summary

The transmissible spongiform encephalopathies (TSEs) are a group of infectious neurodegenerative diseases affecting both humans and animals. The prion hypothesis postulates that prions are protein conformation based infectious agents responsible for TSE infectivity. Prions have been synthetically generated *in vitro*, but it remains unclear whether the properties of synthetically generated prion are the same as those of TSE agents and whether the disease caused by synthetically generated prion is identical to naturally occurring TSEs. In this study, we demonstrated that similar to the classical TSE agents, the synthetically generated prion has a titratable infectivity and is able to cause prion disease in wild-type mice via routes other than direct intra-cerebral inoculation. More importantly, we showed that the synthetically generated prion induced pathological changes, including the dissemination of disease-specific prion protein accumulation and the route and mechanism of neuroinvasion, were all typical of classical TSEs. These results demonstrate the similarity of synthetically generated prion to the infectious agent in TSEs, providing strong evidence supporting the prion hypothesis.

## Introduction

Transmissible Spongiform Encephalopathies (TSEs, also known as prion diseases) are fatal transmissible neurodegenerative disorders, which include bovine spongiform encephalopathy (BSE) in cattle, scrapie in sheep and goats, chronic wasting disease (CWD) in deer and elk, and Creutzfeldt-Jakob disease (CJD) in humans [1,2]. The prion hypothesis postulates that the infectious agent in TSEs is a prion, a misfolded conformer of host encoded normal prion protein (PrP^C^) [3]. Because of its seeding capability, the pathogenic PrP conformer seeds the conversion of host PrP^C^ to more pathogenic PrP form, resulting in prion amplification, accumulation, and neurodegeneration.

Previous studies have established that PrP^C^ and disease specific abnormal PrP are different conformers of the prion protein. PrP^C^ is mainly composed of α-helical structures, soluble in mild detergents and sensitive to protease K (PK) digestion [4–8]. The diseased brains contain various PrP conformers that are different from PrP^C^ [9]. The classical disease specific PrP conformer is rich in β-sheet structures, aggregated and partially resistant to PK digestion [4–8]. The development of more sensitive assays, such as the conformation-dependent immunoassay (CDI) [10], or antibodies specifically recognizing diseased PrP conformers, such as 15B3 antibody [11,12], led to the discovery of various PrP conformers in the diseased brains that are distinct from PrP^C^ [13,14]. Notably, some of these PrP conformers are completely sensitive to PK digestion [13–16] and it was estimated that up to 90% of the disease specific PrP conformers in sporadic CJD cases are PK sensitive PrP conformers [13]. These findings are consistent with the notion that PrP is able to exist in multiple thermodynamically stable conformations [17–19] either by itself or by forming complex with other factors, but whether only one or multiple of these PrP conformers are associated with the prion infectivity remains unclear.

Of all the disease specific conformers, the classical C-terminal PK-resistant PrP conformer, PrP-res, is the best-established disease marker. The association between the presence of PrP-res in tissue homogenates or partially purified PrP-res and prion infectivity, measured by eliciting a prion disease in an animal or chronically infecting susceptible cell lines, is well supported by experimental evidence [1,20]. Despite the fact that the amount of PrP-res did not always correlate with the titer or severity of infectivity [21] and in some cases, prion infectivity was detected without biochemically or pathologically detectable PrP-res [22], PrP-res remains the best biochemically tractable PrP conformer and has been used widely to test the prion hypothesis.

The prion hypothesis explains the infectivity by the seeding capability of disease specific PrP conformers [1,3]. The cell-free conversion assay, in which partially purified PrP-res was used to seed the conformational change of purified PrP^C^, established the seeding capability of PrP-res [23,24]. This conclusion was confirmed by the more efficient serial protein misfolding cyclic amplification (sPMCA) assay [25,26], in which a small amount of diseased tissue homogenate was mixed with large amount of normal brain homogenates and the whole mixture was subjected to successive cycles of sonication and incubation [27]. The sPMCA allows an efficient and almost indefinite replication of the PrP-res conformation from a minute amount of PrP-res seed [28], which led to the demonstration that the PrP-res conformation and prion infectivity are simultaneously propagated via sPMCA [26], establishing a correlation between PrP-res and prion infectivity. Notably, in the absence of PrP-res seed, the PrP-res conformer and prion infectivity were generated by subjecting normal mouse brain homogenates or purified PrP^C^ plus polyanions to sPMCA [29,30], which further strengthens the association between PrP conformational change and the generation of prion infectivity.

Recently, several groups have successfully demonstrated that infectious prions can be synthetically generated from purified bacterially expressed recombinant PrP (recPrP) in biochemically defined *in vitro* systems [31–37]. Although the infectivity varied, these studies have established that purified recPrP can be converted into infectious conformers and cause prion disease in wild-type rodents via intra-cerebral (i.c.) inoculation [31–37].

Despite the advance, it remains unclear whether the rec-Prion share the same pathogenic properties of naturally occurring prions, such as the ability to transmit disease via routes other than the most efficient i.c. inoculation [38–40], the neuroinvasion pathway, and the ability to induce TSE specific lesions. Answers to these questions are important for establishing the prion as a disease-causing agent in TSEs. In this study, we determined the titer of the rec-Prion using end-point titration assays, infected wild-type mice with the rec-Prion via an intraperitoneal (i.p.) route, and performed detailed pathological phenotype analysis. We found that the rec-Prion inoculated mice developed a prion disease with properties, including survival times, pathological lesions, visceral dissemination, and neuroinvasion pathways closely resembling those found for naturally occurring scrapie and CWD.

## Results

### Determining the titer for rec-Prion

We previously showed that highly infectious rec-Prions can be generated *in vitro* by subjecting a substrate mixture consisting of recPrP plus two cofactors, synthetic 1-palmitoyl-2-oleoylphosphatidylglycerol (POPG) and total RNA isolated from normal mouse liver, to sPMCA [31]. The rec-Prion possesses the biochemical hallmarks of the disease specific PrP-res conformer and efficiently causes prion disease in wild-type mice after i.c. inoculation [31]. To determine whether the rec-Prion generated via our protocol contains a titratable infectivity, we performed end-point titration bioassay (Table 1). The rec-Prion inoculum was prepared by sPMCA as previously described [31]. At the end of preparation, the sPMCA product was subject to a 10-fold serial dilution and each diluted sPMCA products was inoculated into wild-type CD-1 mice via an i.c. route. All mice were monitored for the development of prion disease for 500 days post inoculation (dpi). The prion infectivity titer was determined according to the Spearman-Karber formula, which showed that the rec-Prion contained a titer of 10^3.84^ LD_50_ (i.c.) (50% of lethal dose by i.c. route) / μg total rPrP. Thus, we conclude that our sPMCA protocol is able to produce rec-Prion containing an infectivity titer around 10^4^ LD_50_ (i.c.) / μg, confirming that the rec-Prion contains a titratable infectivity.

**Table 1:** End point titration bioassay.

rec-Prion dilution	Survival time (Mean ± SEM)	Number of prion diseased mice / Number of inoculated mice
10^0^	182 ± 5	5/5
10^–1^	180 ± 5	4/4
10^–2^	183 ± 7	4/4
10^–3^	201 ± 26	4/4
10^–4^	219	1/4
10^–5^	> 500*	0/4
10^–6^	260	1/4
10^–7^	> 500**	0/4
10^–8^	> 500	0/4
10^–9^	> 500	0/4

* Two mice died from intercurrent diseases at 147 and 325 dpi.

** One mouse died from intercurrent diseases at 250 dpi.

dpi, days post inoculation; SEM, standard error of the mean

### Intraperitoneal (i.p.) inoculation of rec-Prion in wild-type CD-1 mice

To determine whether rec-Prions can cause disease via routes other than i.c. inoculation, we challenged wild-type CD-1 mice with rec-Prion via an i.p. route. Three negative control inocula were used in this experiment, which were the same controls used in our previous report of i.c. inoculation of rec-Prion [31] (listed in the S1 Table). Control 1, the sPMCA substrate mixture without recPrP, was subjected to the same sPMCA procedure that prepared rec-Prion inoculum. Control 2, the sPMCA substrate mixture, was incubated at 37°C for 24 days without sonication. Control 3 was prepared by directly mixing recPrP, POPG and RNA in inoculum diluent. The amount of each component in control 3 is equal to that in the rec-Prion inoculum.

**Fig 1:**
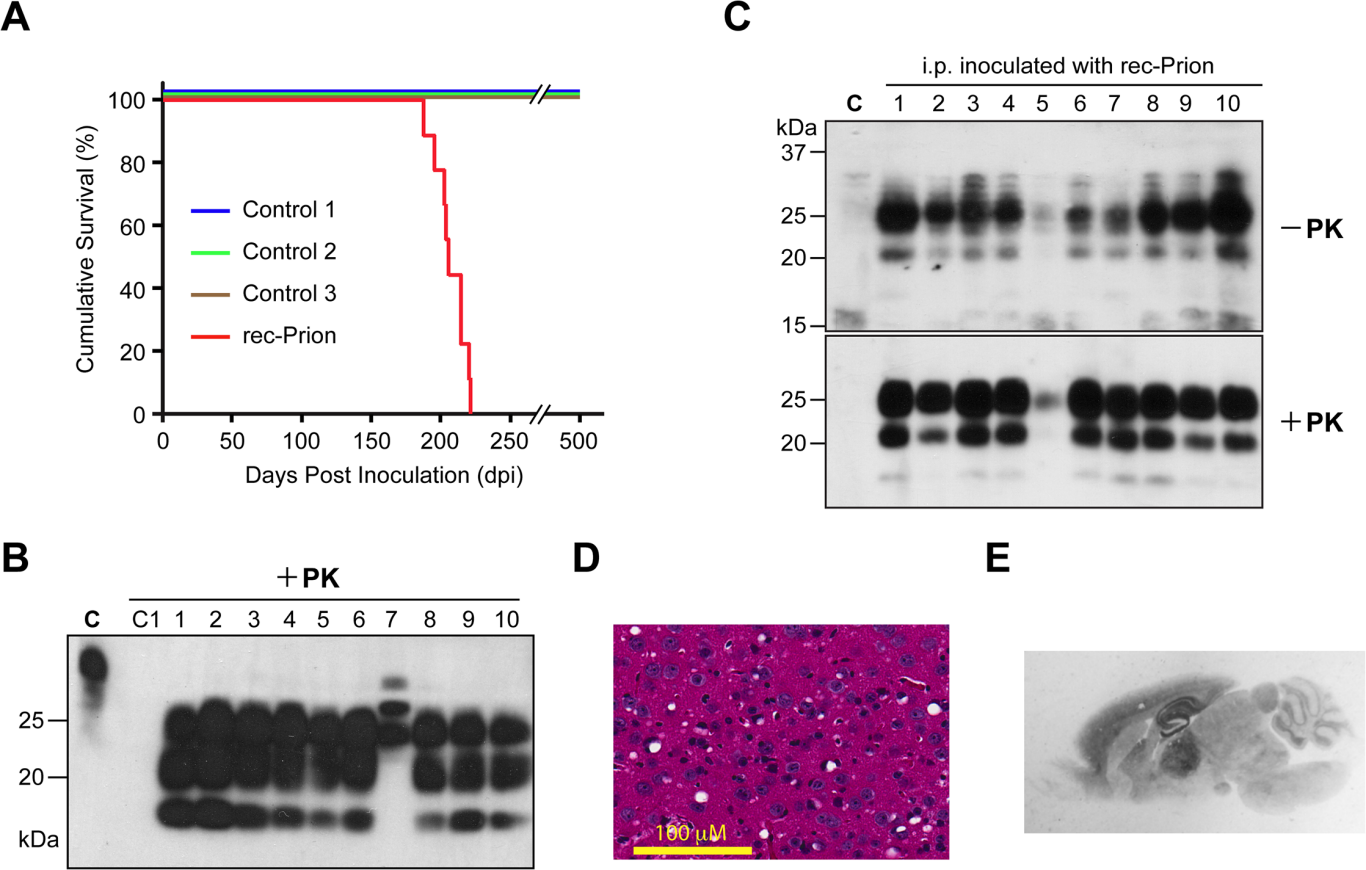
Intraperitoneal inoculation of wild-type CD-1 mice with rec-Prion. **(A)** Survival curves of wild-type CD-1 mice that received i.p. inoculations of rec-Prion or control inocula. **(B)** Immunoblot analysis of PK-resistant PrP in the brain of all 10 mice that received i.p. inoculation of rec-Prion. C, undigested brain homogenate prepared from a rec-Prion inoculated mouse as a control. C1, PK digested brain homogenate prepared from an age-matched, un-inoculated CD-1 mice. PrP was detected by the POM1 monoclonal anti-PrP antibody and a peroxidase conjugated goat anti-mouse IgG (Bio Rad). **(C)** The spleen homogenates prepared from all 10 mice that received i.p. inoculation of rec-Prion were digested with or without PK as indicated. The PrP in the spleen homogenates was detected by the polyclonal M20 anti-PrP antibody (Santa Cruz Biotech. Inc.) and a peroxidase conjugated donkey anti-goat IgG (Santa Cruz Biotech. Inc.). C, homogenate prepared from a spleen of an un-inoculated CD-1 mouse as a control. **(D)** H&E stain of the frontal cortical region of a mouse i.p. challenged with rec-Prion. **(E)** PET blot analysis to show PK-resistant PrP-res deposition in the brain of a mouse received i.p. rec-Prion inoculation.

Except for one mouse (IP#152) that died unexpectedly at 96 days post inoculation (dpi), all other rec-Prion inoculated mice developed clinical signs around 170–200 dpi, including restlessness, hindlimb paralysis, rough coat and weight loss (supporting S1 Fig). The disease progressed rapidly and mice generally reached terminal stage within 2–3 weeks. The average survival time for rec-Prion inoculated mice was 206.8 ± 3.82 dpi (IP#152 mouse was not included). Mice that received control inocula were monitored for 500 dpi and no sign of disease was observed (Fig 1A and Table 2 experiment 1).

**Table 2:** Intraperitoneal transmission studies in wild-type mice.

	Inoculum	Infection Route	Mouse Strain	Attack Rate (N_disease_/N_total_)	Survival Time (dpi) (Mean ± SEM)	Survival Time for Individual Mouse (dpi)
Experiment #1	rec-Prion	i.p.	CD-1	100% (10/10)	206.8 ± 3.82	96*, 187, 195, 202, 203, 205, 214, 214, 220, 221
Experiment #2	IP#152 BH	i.p.	CD-1	20% (1/5)	214	214
	IP#152 BH	i.c.	CD-1	20% (1/5)	219	219
Experiment #3	i.p. BH	i.p.	C57BL/6	100% (5/5)	222.8 ± 5.62	205, 214, 231, 232, 232
	i.p. BH	i.c.	C57BL/6	100% (4/4)	156.3 ± 2.29	151, 154, 159, 161
	i.c. BH	i.p.	C57BL/6	100% (5/5)	214 ± 1.67	208, 213, 215, 217, 217
	i.c. BH	i.c.	C57BL/6	100% (5/5)	160 ± 3.34	151, 154, 166, 166, 166
	rec-Prion	i.p.	C57BL/6	100% (5/5)	220 ± 1.79	213, 221, 221, 222, 223
	rec-Prion	i.c.	C57BL/6	100% (5/5)	172.2 ± 1.11	170,170, 172, 173, 176
Experiment #4	i.c. BH	i.p.	CD-1	100% (5/5)	199.25 ± 10.15	152**, 171, 198, 213, 215
	i.p. BH	i.c.	CD-1	100% (4/4)	184.75 ± 13.20	160, 172, 186, 221
	rec-Prion	i.p.	CD-1	100% (5/5)	217.5 ± 6.41	154**, 200, 217, 223, 230
	rec-Prion	i.c.	CD-1	100% (4/4)	163.5 ± 5.74	154, 158, 162, 180
Experiment #5	rec-Prion	i.p	CD-1	100%	1, 15, 30, 60, 100, 160 (time course study); 210.3 ± 5.33 (terminal disease)	1, 15, 30, 60, 100, 160m (time course study); 205, 205, 221 (terminal disease)

* This mouse (IP#152) developed an intercurrent disease and was culled at 96 dpi.

** These two mice were sacrificed before any clinical signs and their survival times were not included in the calculation of average survival times.

rec-Prion, synthetically generated prion

i.c. BH, 1% brain homogenate prepared from a mouse that received i.c. inoculation of rec-Prion

i.p. BH, 1% brain homogenate prepared from a mouse that received i.p. inoculation of rec-Prion

dpi, days post inoculation

SEM, standard error of the mean

Terminal disease, mice were culled at terminal stage of the disease.

Mouse brain homogenates were subjected to PK digestion and except for the IP#152 mouse, the classic PrP-res signal was detected in all 9 mice that received i.p. rec-Prion inoculation (Fig 1B). The brain of IP#152 mouse contained PK-resistant bands of higher molecular weights (Fig 1B, lane 7), which were confirmed as cross-reacting bands recognized by the secondary antibody (S2 Fig). No PrP-res was detected in the brains of mice that received control inocula. For all 10 mice that were challenged i.p. with rec-Prions, PrP-res was also detected in the spleen (Fig 1C).

Spongiosis (Fig 1D), astrogliosis and microgliosis (S3 Fig) were detected in the brains of 9 i.p. inoculated mice (The IP#152 brain was not available for histological examination). The PK-resistant PrP-res deposition was confirmed by paraffin-embedded tissue (PET) blot (Fig 1E), revealing that PrP-res was deposited widely in the brain and the most prominent deposition was in hippocampus and thalamus, a pattern similar to that of mice that received i.c. rec-Prion inoculation [31]. No spongiosis or PrP-res were detected in the mouse brains inoculated with any of the control inocula (S4 Fig).

### Second round transmission

The detection of PrP-res in the spleen, but not in the brain of IP#152 mouse suggests that it was at an early stage of prion disease and died from an intercurrent disease. Consistent with this notion, second round transmission with IP#152 brain homogenate, either via i.p. or i.c. route, only had an attack rate of 20% (Table 2 Experiment 2; S5 Fig). Nonetheless, the presence of prion infectivity in IP#152 mouse confirmed a 100% attack rate for i.p. rec-Prion challenge in wild-type mice.

Second round transmission of other rec-Prion inoculated mice were performed via i.p. or i.c. route in either C57BL/6 (Table 2, experiment 3) or CD-1 mice (Table 2, experiment 4). As controls, freshly prepared rec-Prions were used to inoculate C57BL/6 or CD-1 mice via either i.c. or i.p. route in all these experiments (Table 2, experiments 3 and 4). All inoculations led to a 100% attack rate. The survival times were 156–172 dpi for i.c. and 214–222 dpi for i.p. inoculation in C57BL/6 mice. In CD-1 mice, the survival times were 163–184 for i.c. and 199–217 for i.p. inoculation.

**Fig 2:**
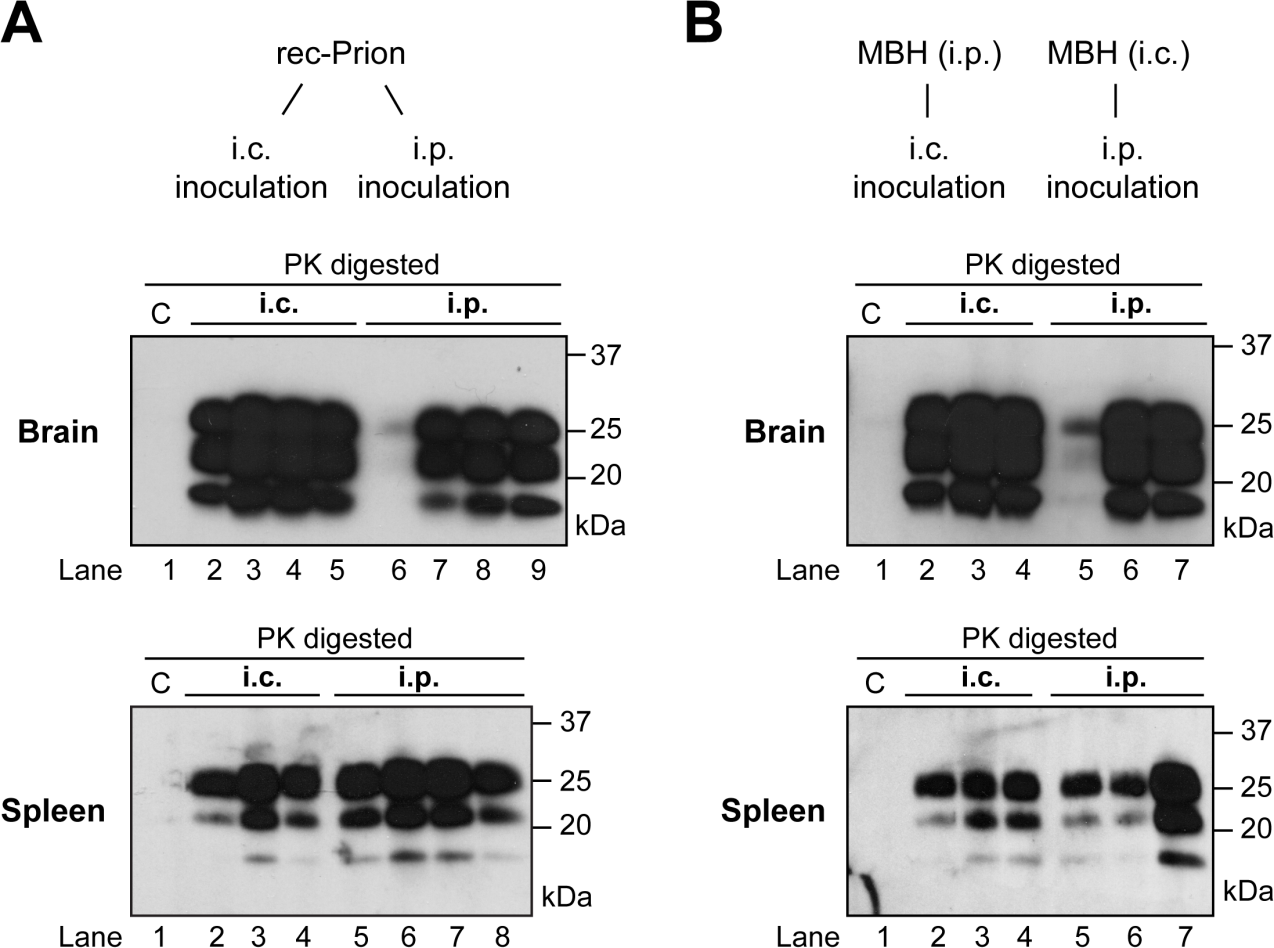
Brain and spleen PrP-res accumulation in rec-Prion inoculated wild-type CD-1 mice. **(A)** PrP-res in brain (top panel) and spleen (bottom panel) of mice that received i.p. or i.c rec-Prion inoculation as indicated. **(B)** PrP-res in brain (top panel) and spleen (bottom panel) of mice that received i.p. or i.c inoculation of mouse brain homogenate (MBH) prepared from mice that received rec-Prion transmission. Two inoculated mice were sacrificed before any clinical signs (154 and 152 dpi), and their PK digested samples were separated in lanes 6 (brain) and 5 (spleen) of **(A)**, and lanes 5 (brain and spleen) of **(B)**. C, PK-digested brain or spleen homogenates prepared from age-matched wild-type CD-1 mice as controls.

All inoculated CD-1 and C57BL/6 mice developed signs of prion disease and accumulated PrP-res (Fig 2A and 2B). Notably, at early stage of the disease, more PrP-res accumulated in the spleen than in the brain (Fig 2A lanes 6 (brain) and 5 (spleen); Fig 2B lanes 5 (brain and spleen). These two mice were sacrificed before clinical signs became apparent, suggesting that PrP-res may accumulate first in the spleen. The fact that spleen PrP-res accumulation was detected in both i.p. and i.c. challenged mice (Fig 2) suggests that the rec-Prion is a lymphotropic prion, which is able to replicate and accumulate in peripheral lymphoid organs. The accumulation of PrP-res in lymphoid organs following i.c. rec-Prion inoculation provides a disease phenotype that is similar to that of naturally occurring contagious prions [41–44].

### Pathology of rec-Prion caused disease

**Fig 3:**
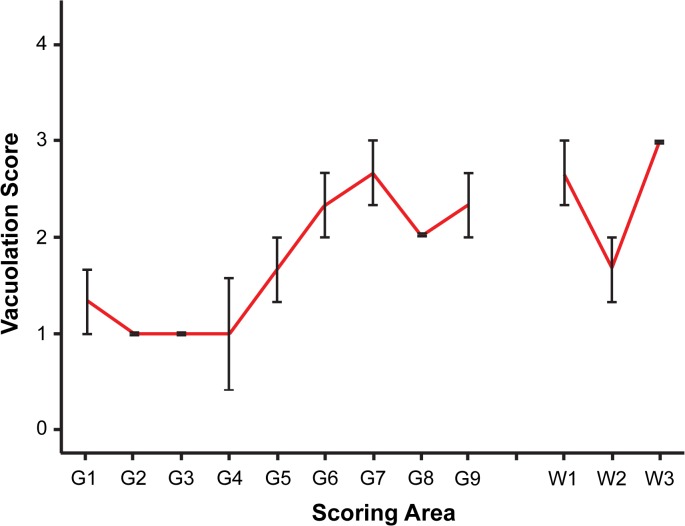
Vacuolar lesion profile of CD-1 mice challenged by i.p. inoculation of rec-Prions. Spongiosis in the following brain regions were scored. Grey matter regions: G1, dorsal medulla; G2, cerebellar cortex; G3, superior colliculus; G4, hypothalamus; G5, medial thalamus; G6, hippocampus; G7, septum; G8, parietal cerebral cortex; G9, frontal cerebral cortex. White matter regions: W1, cerebellar white matter; W2, mesencephalic tegmentum; W3, pyramidal tract. Error bars represent SEM (standard error of the mean).

**Fig 4:**
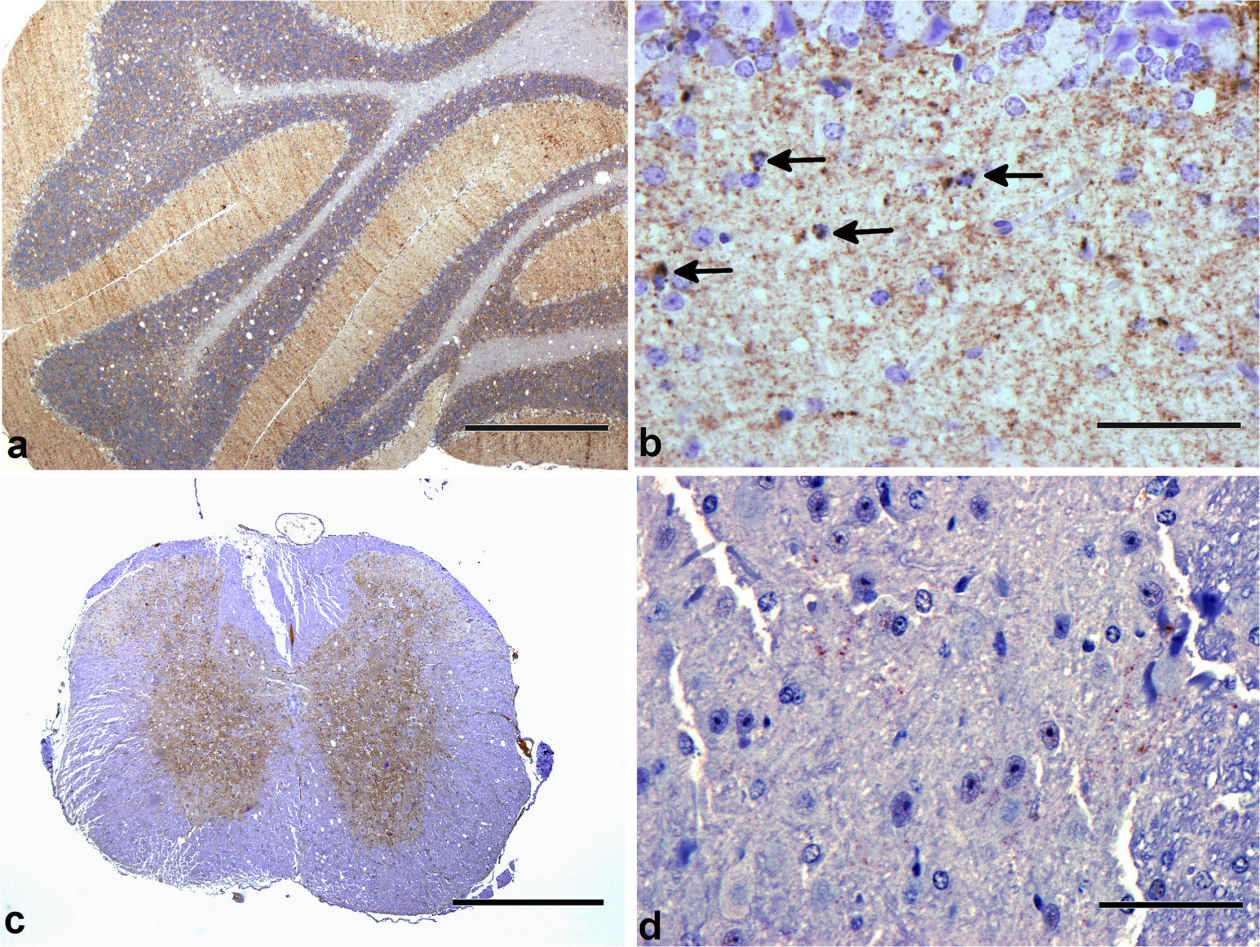
PrP-d accumulation in CNS. Patterns of PrP-d accumulation in brains of wild-type CD-1 mice i.p. challenged with rec-Prions. **(a)** Cerebellum at terminal disease stage showing diffuse PrP-d accumulations in the molecular and granule cell layers, and moderate vacuolation of white matter and granule cell layers. Bar = 500 μm. **(b)** Cerebellum at higher magnification showing intracellular accumulation of PrP-d in glial cells (arrows) and diffuse punctuate nature of accumulation in the molecular layer. Bar = 50 μm. **(c)** Cervical spinal cord showing diffuse PrP-d accumulation throughout grey matter (and moderate white matter vacuolation). Bar = 500 μm. **(d)** Thoracic spinal cord showing sparse punctuate PrP-d accumulation in the intermediolateral column of a mouse sacrificed at 100 dpi. Bar = 50 μm.

The ability to infect mice via the i.p route allows us to determine the pathogenic changes in rec-Prion caused disease, including disease pathology in central nervous system (CNS) and lymphoreticular system (LRS), and the route and mechanism of neuroinvasion. Detailed pathological analyses and a time course study were performed (Table 2 experiment 5), which allows us to compare the i.p. rec-Prion challenge induced disease to that of classical rodent scrapie. Figs 3 and 4 shows the patterns of vacuolation and immunohistochemically detected aberrant PrP deposition (PrP-d, the disease specific PrP form detected by histological methods) in terminally diseased mice. PrP-d in CNS and spinal cord was mainly found as diffuse punctuate forms with frequent granular intra-cellular accumulations (Fig 4) and occasional small plaque-like deposits. Thus, both the nature and patterns of vacuolation and PrP-d accumulation are consistent with those found in different strains and isolates of laboratory murine scrapie and supports the contention that rec-Prions causes *bona fide* prion disease in rodents [31].

**Fig 5:**
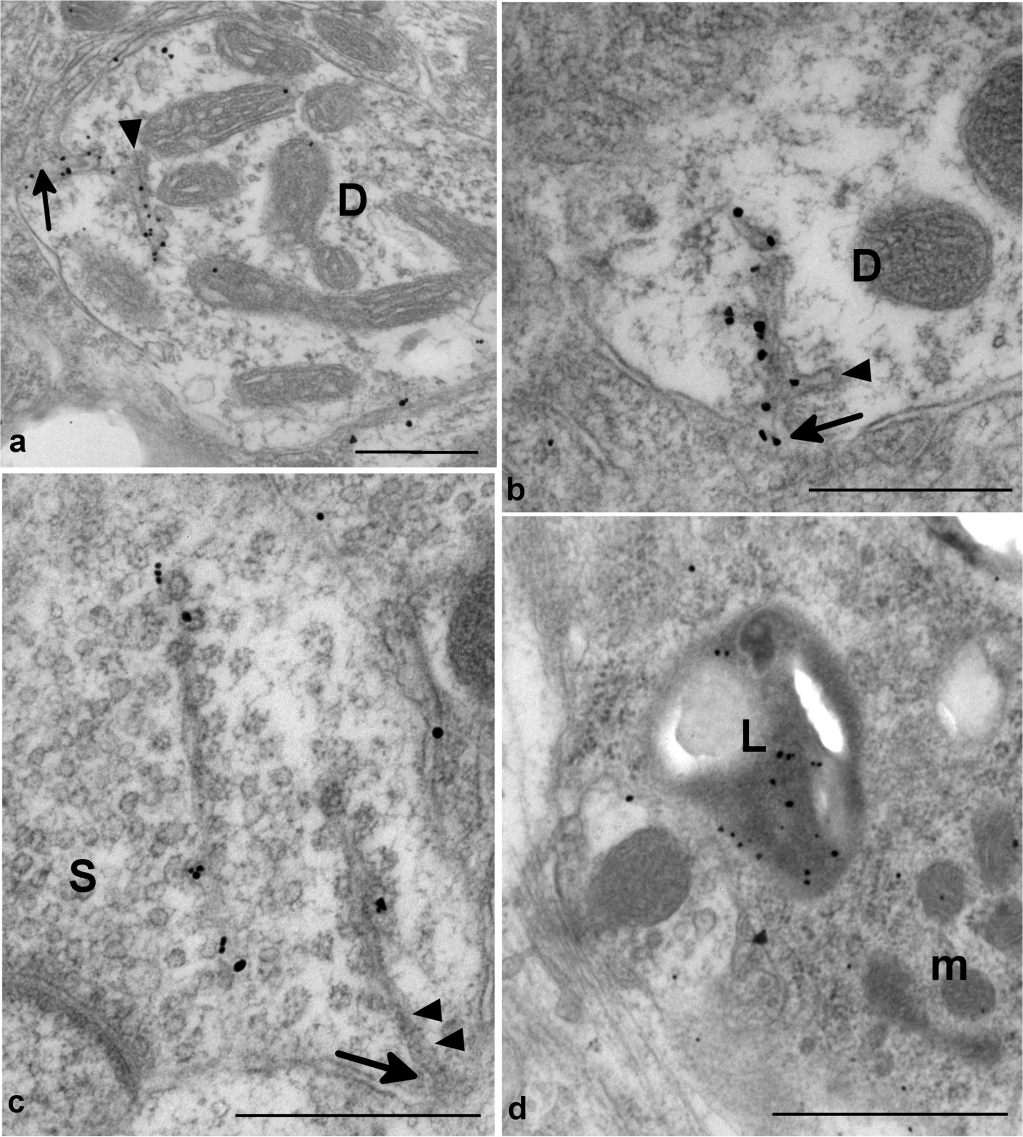
Ultrastructural changes in rec-Prion challenged CD-1 mice detected by immunogold electron microscopy. **(a)** and **(b)** show dendritic membrane invaginations (arrows) in two dendrites (D). The reflected membrane invagination is labelled for PrP-d. The membrane invaginations are branched and the ends of the branches show a membrane coating on the cytoplasmic face of the membrane (arrowheads). Bars = 500 nm. **(c)** shows two weakly PrP-d labelled spiral membrane invaginations with continuous membrane undercoated buds in an axon terminal containing numerous synaptic vesicles (S). The spiral nature of the invaginations can be seen between arrowheads. One invagination is tangentially sectioned and lies apparently free within the cytoplasm. The other is visibly connected to the membrane (arrow). Bar = 500nm. **(d)** shows immunogold labelling for PrP-d within a lysosome (L) of a microglial process (m). Bar = 1 μm. All images were taken from medulla of i.p. challenged terminal disease affected mouse and 1A8 antibody was used to perform the immunogold detection of PrP-d.

Classical forms of prion disease in rodents, farm animals and man are associated with highly specific PrP-d co-localized ultrastructural membrane changes and intra-lysosomal PrP-d accumulation [13,45]. These features are characteristic and unique to prion disease. Using immunogold electron microscopy, we confirmed these distinctive features in rec-Prion challenged mice examined at terminal stage disease (Fig 5), including PrP-d associated membrane ruffling, branched membrane invaginations with sub-membrane coating on the ends of branches (Fig 5A and 5B) and spiral membraneinvaginations with continuous coating of spiral membranes and buds (Fig 5C). The membrane invaginations were encountered on both dendrites and axon terminals. Lysosomal PrP-d accumulation was also identified in glia (Fig 5D) and neurons. These ultrastructural pathological features confirm the phenotypic similarity of rec-Prion caused disease with classical prion diseases.

**Fig 6:**
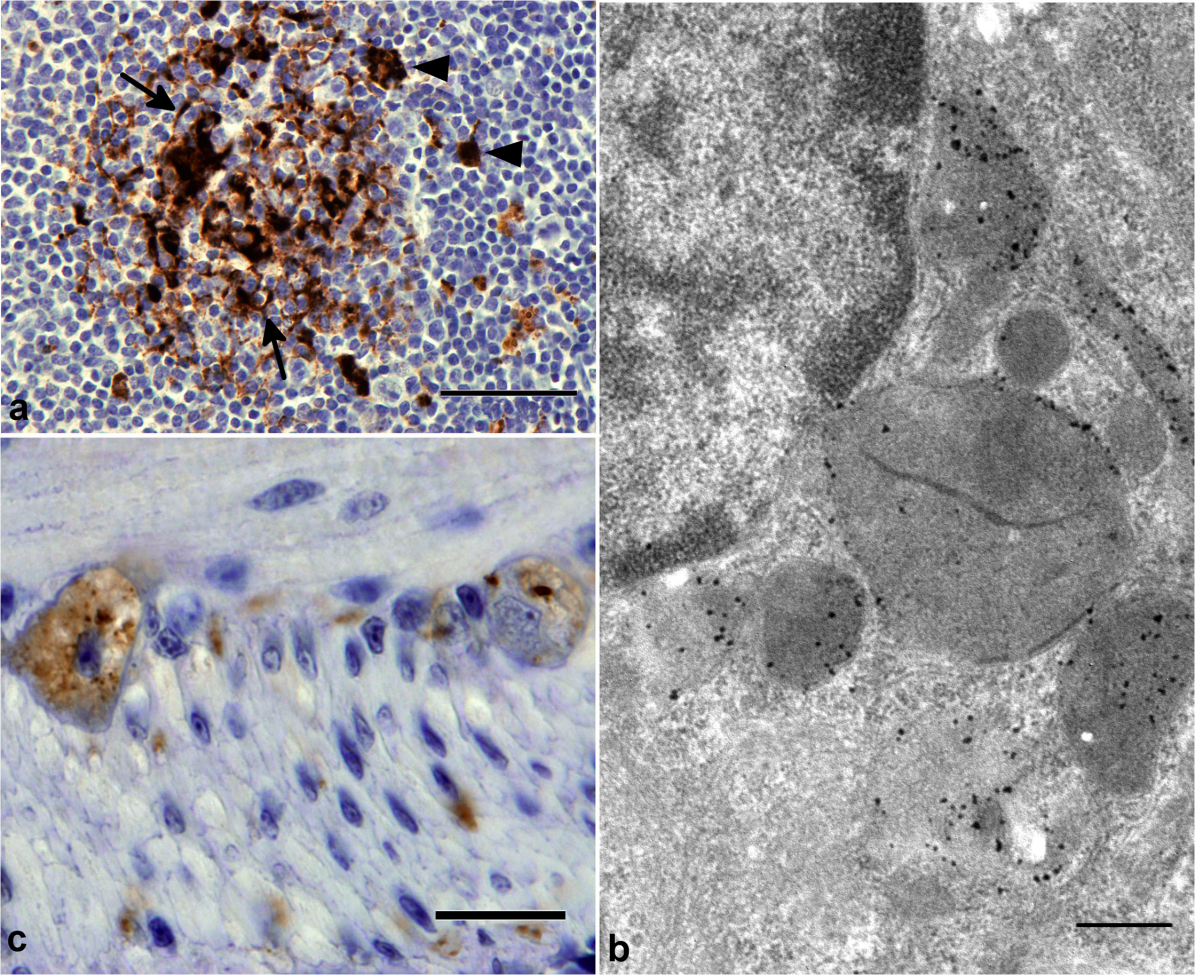
PrP-d accumulation in viscera of CD-1 mice i.p. challenged with rec-Prions. **(a)** Terminal disease lymph node tissue showing granular PrP-d accumulation in Tingible body macrophages (TBMS, arrowheads) and diffuse punctuate PrP-d accumulation in association with follicular dendritic cells (arrow). Bar = 50μm. **(b)** Immunogold electron microscopy of terminal disease lymph node shows PrP-d (black) in lysosomes and endo-lysosomes of TBMs. The 1A8 antibody was used for immunogold staining. Bar = 500 nm. **(c)** Terminal disease intestine showing granular PrP-d accumulation in neurons of enteric nerve plexuses. Bar = 20 μm.

In lymphoid tissues PrP-d was confined to germinal centers of lymph nodes, Peyer’s patches of the intestine and spleen where it was associated with both tingible body macrophages (TBMs) and follicular dendritic cells (Fig 6). Immunogold electron microscopy localized PrP-d to the lysosomes of TBMs (Fig 6) and the cell membranes of the dendrites of follicular dendritic cells. In the enteric nervous system PrP-d was in the cytoplasm of enteric neurons and satellite cells (Fig 6).

Collectively, the patterns of cellular and sub-cellular localization of PrP-d in i.p. rec-Prion challenged mice in the CNS, LRS and in the enteric nervous system (ENS) are indistinguishable from classical forms of experimental rodent prion disease and of naturally occurring contagious TSEs [46], confirming the diagnosis of rec-Prion caused disease as a TSE or prion disease.

### Pathogenesis of i.p. rec-Prion challenge caused prion disease

A time course study was performed to determine the sequential pathogenic changes caused by rec-Prion. The temporal sequence of the spread was determined by immunohistochemistry on selected viscera and CNS tissues at 1, 15, 30, 60, 100, 160 dpi, and terminal disease stages (Table 3). PrP-d accumulation was first detected in lymphoid tissues at 30 dpi, while the first PrP-d accumulation in the CNS were found at 100 dpi (Table 3). In spinal cord at 100 dpi, sparse puncta of PrP-d was confined to the intermediolateral column of the thoracic spinal cord (Fig 4) in 3 of 5 mice, but not the cervical or lumbar spinal cord. (In the other 2 mice, the PrP-d distribution in spinal cord was more widespread at 100 dpi.) The first PrP-d accumulation in the brain was found in white matter tracts of the medulla and midbrain. Thus the initial sites and dissemination of PrP-d in spinal cord are consistent with previous oral and natural prion disease pathogenesis studies in laboratory rodents [47] and in sheep [48,49], suggesting neuroinvasion ascended to this site via autonomic nerves of the abdomen.

**Table 3:** Temporal sequence of PrP-d accumulation in various tissues after i.p. rec-Prion inoculation (PrP-d positive samples / Total samples examed).

Tissue Compartments	Days post i.p. inoculation						
	1	15	30	60	100	160	Terminally diseased
CNS	0/5	0/5	0/5	0/4	5/5	4/4	3/3
Spinal Cord	0/5	0/5	0/5	0/4	5/5	4/4	3/3
LRS	0/5	0/5	3/5	4/4	5/5	4/4	3/3
PNS (ENS)	0/5	0/5	0/5	0/4	0/5	2/4	3/3

PrP-d, disease specific aberrant PrP deposition

CNS, central nervous system

LRS, lymphoid tissues

PNS (ENS), peripheral autonomic system (enteric nervous system)

PrP-d was initially detected in the lymphoid tissues of 3 out of 5 mice at 30 dpi (Tables 3 and 4). By 60 dpi PrP-d accumulation was detected in almost all LRS tissues (Table 4), but not in the ENS. The most conspicuous early amplification occurred in the spleen at 30 dpi and affected most lymphoid tissues by 60 dpi. The rapid involvement of all LRS tissues combined with the absence of any physical connection between them argues for a hematogenous dissemination of infectivity at a relatively early stage of infection, which is consistent with serial pathogenesis studies of sheep scrapie and BSE [48–50]. The first detection of PrP-d in the ENS occurred at 160 dpi and affected all mice only at terminal stage disease (Table 3), suggesting a centrifugal spread of PrP-d from CNS.

**Table 4:** Temporal sequence of PrP-d accumulation in lymphoid tissues after i.p. rec-Prion inoculation (PrP-d positive samples / Total samples examed).

LRS tissues	Days post i.p. inoculation						
	1	15	30	60	100	160	Terminally diseased
Spleen	0/4	0/5	3/5	4/4	5/5	4/4	3/3
SMLN	0/4	0/5	0/5	4/4	5/5	4/4	3/3
lgLN	0/4	0/5	2/5	4/4	5/5	4/4	3/3
MLN	0/4	0/5	2/5	4/4	4/4	4/4	3/3
PP	0/0	0/2	0/4	2/4	5/5	4/4	3/3

PrP-d, disease specific aberrant PrP deposition

SMLN, submandibular lymph node

lgLN, inguinal lymph node

MLN, mesenteric lymph node

PP, Peyer’s patches

## Discussion

In this study, we showed for the first time that i.p. inoculation of rec-Prion is sufficient to cause prion disease in wild-type mice. Moreover, detailed pathological analyses confirmed that the characteristics of rec-Prion caused disease are consistent with naturally occurring contagious TSEs. Together with other properties of rec-Prion demonstrated in previous studies [31,34], our data support the concept that infectivity in TSEs can be the result of a protein conformation based infectious agent.

In natural sheep scrapie, CWD of deer and following experimental oral challenge with these agents in laboratory rodents, prion infectivity is found in CNS, LRS tissues and the autonomic nervous systems [46,51]. In naturally occurring contagious TSEs and following experimental oral challenge, infectivity of the gut associated lymphoid tissues precedes generalized hematogenous distribution to other lymphoid tissues [48,51]. Mice i.p challenged with rec-Prions in the present experiments also showed generalized involvement of LRS tissues at a relatively early stage of disease, but in contrast to naturally contagious TSEs, rec-Prion challenged mice showed an early accumulation in spleen and a relatively delayed accumulation in Peyer’s patches (Table 4). This pattern of distribution likely reflects an initial dispersal of the inoculum by peritoneal macrophages rather than intestinal trans-epithelial absorption of infectivity such as that occurs following oral exposure [52].

Following oral or natural exposure [47,49], PrP-d in CNS tissues is first detected in the intermediolateral column of the thoracic spinal cord and the dorsal parasympathetic nucleus of the vagal nerve (DMNV) in the brain medulla, sites corresponding to neuroinvasion by ascending infection of autonomic nerves of the abdomen and intestine respectively [47–49]. Neuroinvasion in the spinal cord of rec-Prion challenged mice also corresponds to an ascending infection via abdominal autonomic nerves. However there was no specific involvement of the DMNV at any stage suggesting a single site of neuroinvasion by rec-Prions and corresponding to late involvement of Peyer’s patches. The initial PrP-d location in the medulla and midbrain probably reflects an ascending infection from the spinal cord. Thus, i.p. inoculated rec-Prions have more closely replicated naturally occurring scrapie and CWD, insofar as the nature of rapid hematogenous dispersal of infectivity through LRS tissues and ascending neural infectivity found in the present experiments are in agreement with naturally contagious TSEs.

Prions are similar to viruses insofar as their infectivity can be determined by end-point titration. Here we showed that the titer of rec-Prion generated by our methodology [31,34,53] can be determined by end-point titration. It is important to note that sPMCA is influenced by several uncontrollable factors, particularly the stochastic events during the sonication step, which may cause the titer of each rec-Prion batch to vary. Nonetheless, our result does show that end-point titration can be used to titrate the infectivity associated with rec-Prion and our protocol is able to generate an infectivity around 10^4^ LD_50_ (i.c.) / μg. These values are lower than those obtained for some highly adapted experimental rodent strains [54], but are only one log lower than those found for some highly pathogenic sources such as experimental transmission of BSE in sheep and mice [55].

Numerous naturally occurring and laboratory strains of prion disease can be recognized in sheep [56,57] and laboratory rodents [58]. More than 20 strains of scrapie have been identified in mice using selected properties of disease [58,59]. The mechanisms by which a misfolded protein can code for these strain specific properties is unclear, but it has been postulated that tertiary and/or quaternary conformation of the prion may carry such information [1]. The disease phenotype of rec-Prion has minor difference from other commonly used laboratory strains of scrapie, in particular the white matter vacuolation was unusually prominent [60]. The nature of PrP-d deposition and low abundance of plaques readily distinguishes the rec-Prion disease generated here from other diseases initiated by inoculation of misfolded synthetic PrP generated by other means [61,62]. These data, and the absence of disease in mice challenged with control inocula, provide confidence that the present disease transmissions were not caused by laboratory contamination.

The rec-Prion was generated several times in the present study and the disease phenotype, insofar as it was characterized in each experiment, was relatively constant, indicating that our method of producing rec-Prion may constrain the nature of rPrP misfolding process. The rec-Prion seed used in our sPMCA may guide the misfolding of recPrP. In addition, the cofactors used in our protocol, both of which alter recPrP conformation [63–67], may limit the options available to recPrP for misfolding. Thus, instead of a stochastic misfolding process, the recPrP aggregate generated with our method may follow rather limited misfolding possibilities, resulting in a rather constant disease phenotype in mice.

Collectively, our study showed for the first time that rec-Prions are able to cause *bona fide* prion disease in wild-type mice not only through i.c. inoculation, but also via the i.p. route. Moreover, our results established that rec-Prion caused disease shares the pathogenic and pathologic properties with rodent adapted prions and naturally occurring contagious scrapie and CWD, confirming that rec-Prion-caused disease is a prion disease. The relatively simple, yet powerful *in vitro* system to generate rec-Prion will help us to elucidate the molecular mechanism of prion infectivity in future studies.

## Materials and Methods

### Mouse bioassay

For end-point titration, the sPMCA product was 10-fold serially diluted with the inoculation diluent (PBS containing 1 mg BSA/mL). Thirty microliter of inoculum, undiluted or diluted sPMCA product, was inoculated into a mouse via an i.c. route. The i.c. injection, animal monitoring, biochemical and histopathological analyses were performed as previously described [31,53].

For i.p. inoculation, the inoculum was prepared as previously described [31,53]. Briefly, the sPMCA product was washed twice with PBS through pelleting-and-resuspension, and the final pellet was resuspended in the inoculating diluent. The volume of final resuspension was 1/10 (experiment 1) or 1/1 (other experiments 2, 3, 4 and 5) of the original volume of the sPMCA product. Each mouse was intraperitoneally injected with 50 μL of the inoculum. The inoculated mice were monitored three times a week. Once neurological signs were clearly identified, mice were monitored daily and euthanized at terminal disease stage. Second round transmission was performed as previously described [31,53].

### Ethics statement

This study was carried out in accordance with the recommendations in the Guide for the Care and Use of Laboratory Animals of the National Institutes of Health. The protocols were approved by the Institutional Animal Care and Use Committees of the Van Andel Research Institute (Assurance Number A4383-01) and the Ohio State University (Assurance Number A3261-01).

### *In vitro* rec-Prion preparation

For experiment 1, the expression and purification of recPrP and the conditions for sPMCA were exactly the same as originally described [31,53]. For other experiments (Table 2), expression and purification of recPrP and the sPMCA protocol were slightly modified [34]. Briefly, the sPMCA was carried out with a substrate mixture containing 25 μg/mL recPrP (recPrP concentration was determined by OD_280_ reading and the Є280 molar extinction coefficient of recombinant murine PrP 23–230 with one disulfide bond), 22.2 μg/mL POPG (Avanti Polar Lipids), and 150 μg/mL total RNA isolated from normal mouse liver (RNA concentration was determined by OD_260_ reading). The sPMCA substrate was prepared as previously described [31,53]. One round of sPMCA consists of 48 cycles of 30-second sonication followed by 29.5-minute incubation at 37°C. At end of each round, 1/10 sPMCA product was transferred to a fresh tube containing 90 μL of sPMCA substrate and then proceeded to a new round of sPMCA. All rec-Prion inocula used in this study were prepared with seeded sPMCA, which are 1–3 rounds of sPMCA seeded with previously generated rec-Prions.

The recPrP purification in experiment 1 was exactly the same as originally described [31,53]. For recPrP purification in other experiments, *E*. *Coli* BL21 (DE3) cells were transformed with the pET-22b-moPrP23-230, which expresses the un-tagged version of murine PrP 23–230. Detailed protocol for purifying untagged recombinant murine PrP23-230 is described in the supporting S1 Method.

### Pathological analyses

In Experiments 1–4 brains were removed from the calvarium, sagittally sectioned and half brains frozen for subsequent immunoblotting. The remaining half brains were embedded in paraffin wax and stained by HE, immunohistochemistry, and PET blot as previously described [31,53,68]. From experiment 5, brains, five lymphoid tissues (spleen, mesenteric lymph node, inguinal lymph node, sub-mandibular lymph node) and four samples of intestine, including ileum with Peyer’s patches, were fixed in formalin. Brains were coronally sectioned at 2–3 mm intervals and processed through paraffin wax for haematoxylin and eosin staining. Vacuolar lesion profiles were constructed on nine grey, and three white matter sites according to standard methods [69]. Sections were also immunolabelled for PrP-d using the routine methods [70]. The antibodies SAF84, 1C5 and 2G11 were used on each section. All illustrated results are from the antibody 2G11 that gave the least background results.

### Ultrastructural immunohistochemical labelling

Additional terminally diseased mice were perfusion fixed using 4% paraformaldehyde / 0.1% gluteraldehyde for ultrastructural analysis. Light microscopical resin immunolabelling — Selected brain areas (cerebellar cortex, medulla and midbrain), mesenteric and inguinal lymph nodes of 2 rec-Prion inoculated mice and 2 control mice (i.p. inoculated with sPMCA substrate) were processed into epoxy resin as described previously [71] and sections cut at 1 μm. The avidin-biotin complex immunohistochemical staining method was applied to the etched and pre-treated 1 μm sections using 1C5 PrP antibody at a 1:100 or 1A8 antibody at 1:2000 dilution. Sections were examined by light microscopy and selected blocks with appropriate immunolabelled follicles and control blocks were then taken for sub-cellular studies. The method employed in the study of TSE pathology in resin embedded tissues does not show any PrP^C^ labelling in control tissues, therefore PrP detected in these tissues is by definition, disease associated. Immunolabelling was consistently found to be superior in mesenteric lymph nodes therefore only this lymph node was taken for further EM analysis. Multiple sections from at least 2 blocks containing follicles were studied from each animal. Ultrastructural immunohistochemical labelling — 65 nm sections were taken from resin blocks previously found to show PrP-d labelling (or controls containing follicles) and immunolabelled as described previously [71]. PrP-d was detected using primary antibody 1C5 at a 1:10 or 1A8 at 1:500 dilution in incubation buffer. A pre-immune serum was used as a control. At least two 1mm^2^ sections were studied from each animal.

### PK digestion and immunoblot analysis

PK digestion and immunoblot analysis were performed similarly as previously described [31,68]. For analyzing PrP-res in mouse tissues, 20 μg brain homogenate or 40 μg spleen homogenate were subjected to 50 μg / mL PK digestion at 37°C for 1 hour. PrP-res was detected by immunoblot analysis with the POM1 anti-PrP antibody [72] (for brain) or M20 polyclonal anti-PrP antibody (Santa Cruz Biotechnology, Inc.) (for spleen).

### Anti-PrP antibodies

The following antibodies were used for detecting PrP in this study. The SAF84 monoclonal antibody recognizing an epitope of residue 160–170 of hamster PrP, and the 2G11 monoclonal antibody raised against a synthetic peptide (146–182) of the ovine PrP were purchased from Cayman Chemical. The M20 antibody (Santa Cruz Biotechnology, Inc.) is a purified goat polyclonal antibody raised against C-terminus of mouse PrP. The 1C5 antibody recognizes an epitope of residue 119–130 of hamster PrP [73]. The 1A8 is a polyclonal antibody against PrP [74,75]. POM1 recognizes a discontinuous epitope at C-terminus of PrP [72,76].

## Supporting Information

S1 Fig Terminally diseased CD-1 mice i.p. challenged with rec-Prions. (TIF)

S2 Fig PK digestion and immunoblot analyses of brain homogenates prepared from IP#152 mouse. **A.** IP#152 mouse brain homogenate (right) was subject to PK digestion at 37°C for 1 hour with increased PK concentrations as indicated. Brain homogenate prepared from a control mouse was used as a control for the PK digestion (left). **B.** Brain homogenates prepared from a control CD-1 mouse (C), IP#152 mouse (IP152), and IP#151 mouse (IP151) were subject to PK digestion and immunoblot analysis. The blot on the left panel was incubated with POM1 anti-PrP monoclonal antibody and a peroxidase conjugated goat anti-mouse IgG secondary antibody. The blot on the right panel was incubated only with the peroxidase conjugated goat anti-mouse IgG secondary antibody. The detection of the PK-resistant bands in IP#152 by secondary antibody indicated that these bands were mouse IgG.
(TIF)

S3 Fig Immunohistochemical staining with anti-GFAP or anti-Iba1 antibody. Brain sections prepared from un-inoculated CD-1 mice (Control) or terminally diseased CD-1 mice i.p. challenged with rec-Prion (rec-Prion (i.p.)) were stained with an anti-GFAP antibody (astroglia marker) or with an anti-IbaI antibody (microglia marker) as indicated. Dark brown stain represents positive stain, which revealed astrogliosis and microgliosis in the brains of terminally diseased animals.
(TIF)

S4 Fig No spongiosis or PrP-d was detected in CD-1 mice received i.p. inoculation of control inocula. **A.** H&E stain of frontal cortical regions of wild-type CD-1 mice received i.p. inoculation of control inoculum 1, 2 or 3 (listed in S1 Table) as indicated. Bar: 100 μM. **B.** PET blot analysis of wild-type CD-1 mice received i.p. inoculation of control inoculum 1, 2 or 3 as indicated.
(TIF)

S5 Fig PK digestion of brain homogenates prepared from mice received i.c. or i.p. inoculation of brain homogenate prepared from IP#152 mouse. Brain homogenates prepared from wild-type CD-1 mice that received i.c. or i.p. inoculation of IP#152 mouse brain homogenate were subjected to PK digestion followed by immunoblot analysis. PrP was detected with POM1 anti-PrP antibody. The mice without any clinical signs were sacrificed after > 500 dpi. C, undigested mouse brain homogenate used as a control.
(TIF)

S1 Method The rPrP purification protocol. (DOCX)

S1 Table Controls for Experiment 1. (DOC)
