# Supplementary figures and images for: Intraperitoneal Infection of Wild-Type Mice with Synthetically Generated Mammalian Prion
Source: PLoS Pathog. 2015 Jul 2;11(7):e1004958. doi: 10.1371/journal.ppat.1004958 (PMC4489884; doi:10.1371/journal.ppat.1004958)

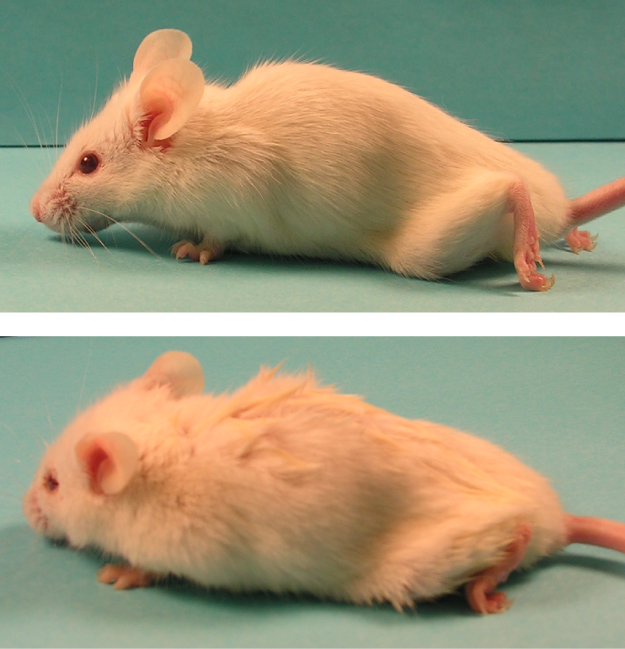

Supplement: S1 Fig — (TIF) [file ppat.1004958.s001.tif]

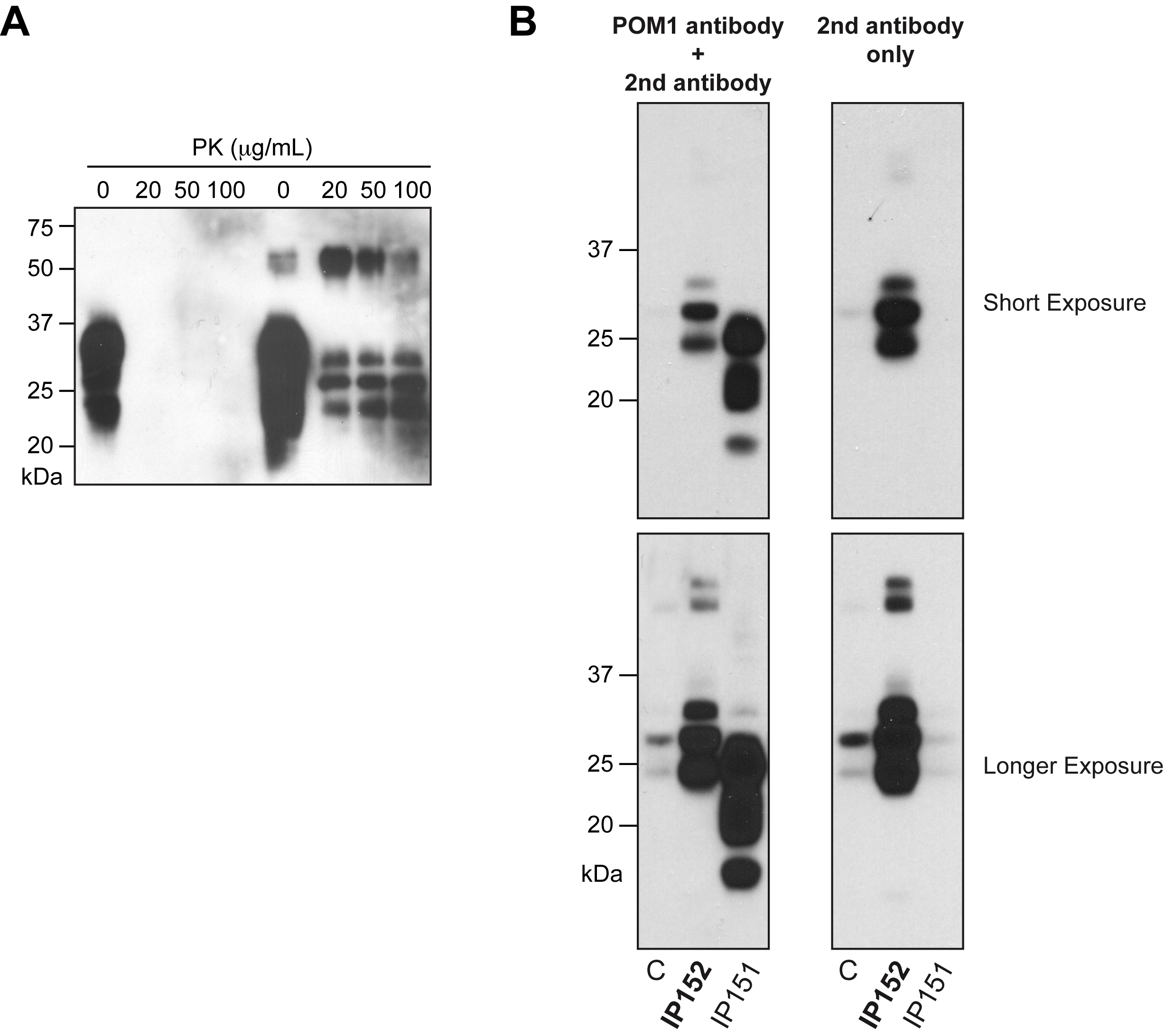

Supplement: S2 Fig — A. IP#152 mouse brain homogenate (right) was subject to PK digestion at 37°C for 1 hour with increased PK concentrations as indicated. Brain homogenate prepared from a control mouse was used as a control for the PK digestion (left). B. Brain homogenates prepared from a control CD-1 mouse (C), IP#152 mouse (IP152), and IP#151 mouse (IP151) were subject to PK digestion and immunoblot analysis. The blot on the left panel was incubated with POM1 anti-PrP monoclonal antibody and a peroxidase conjugated goat anti-mouse IgG secondary antibody. The blot on the right panel was incubated only with the peroxidase conjugated goat anti-mouse IgG secondary antibody. The detection of the PK-resistant bands in IP#152 by secondary antibody indicated that these bands were mouse IgG. (TIF) [file ppat.1004958.s002.tif]

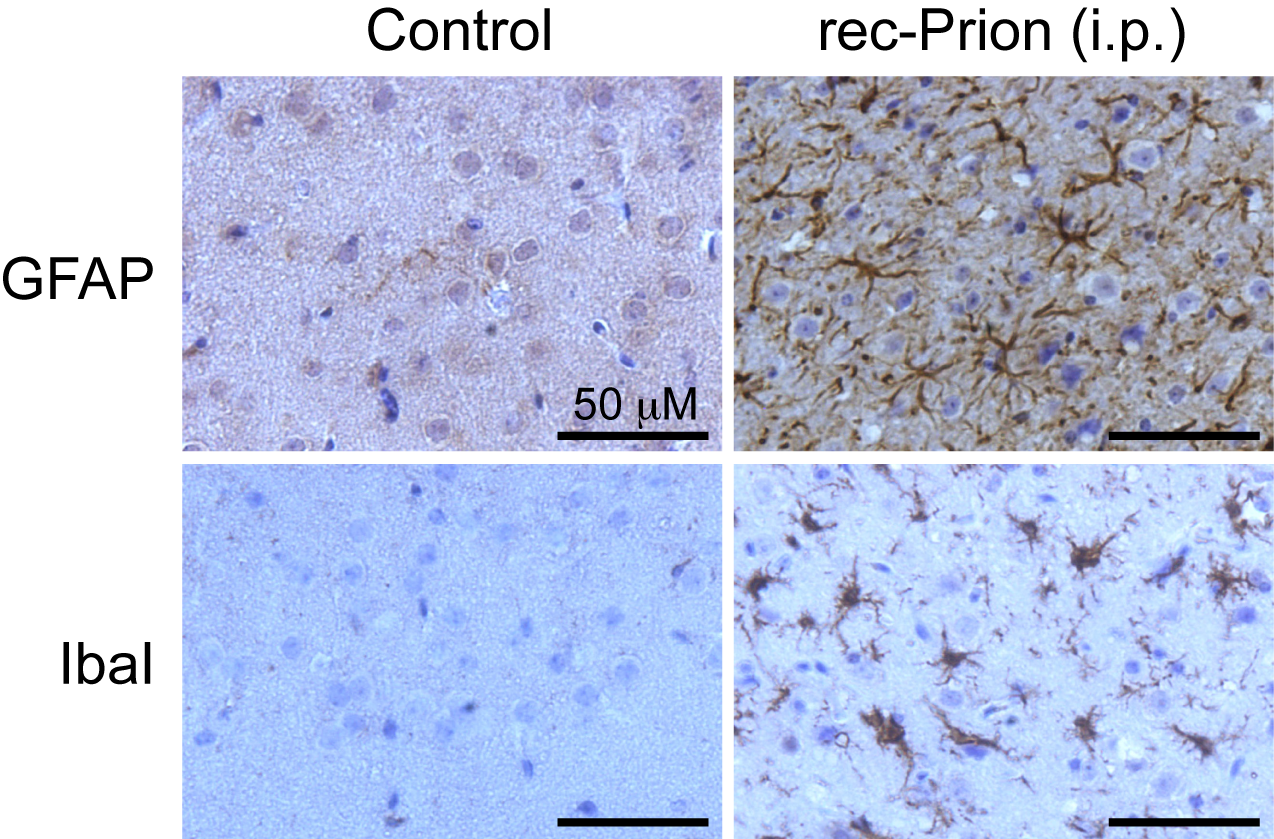

Supplement: S3 Fig — Brain sections prepared from un-inoculated CD-1 mice (Control) or terminally diseased CD-1 mice i.p. challenged with rec-Prion (rec-Prion (i.p.)) were stained with an anti-GFAP antibody (astroglia marker) or with an anti-IbaI antibody (microglia marker) as indicated. Dark brown stain represents positive stain, which revealed astrogliosis and microgliosis in the brains of terminally diseased animals. (TIF) [file ppat.1004958.s003.tif]

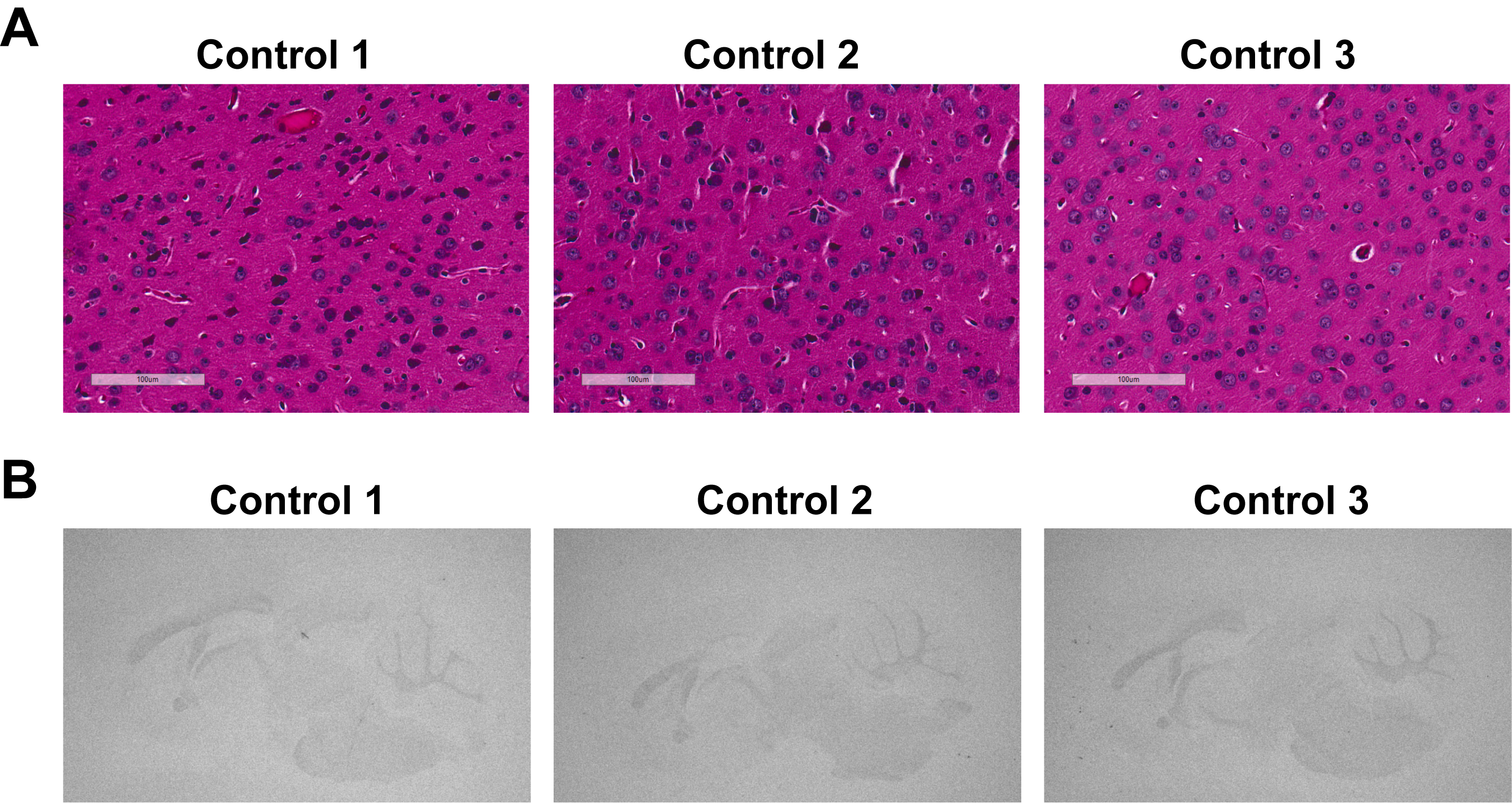

Supplement: S4 Fig — A. H&E stain of frontal cortical regions of wild-type CD-1 mice received i.p. inoculation of control inoculum 1, 2 or 3 (listed in S1 Table) as indicated. Bar: 100 μM. B. PET blot analysis of wild-type CD-1 mice received i.p. inoculation of control inoculum 1, 2 or 3 as indicated. (TIF) [file ppat.1004958.s004.tif]

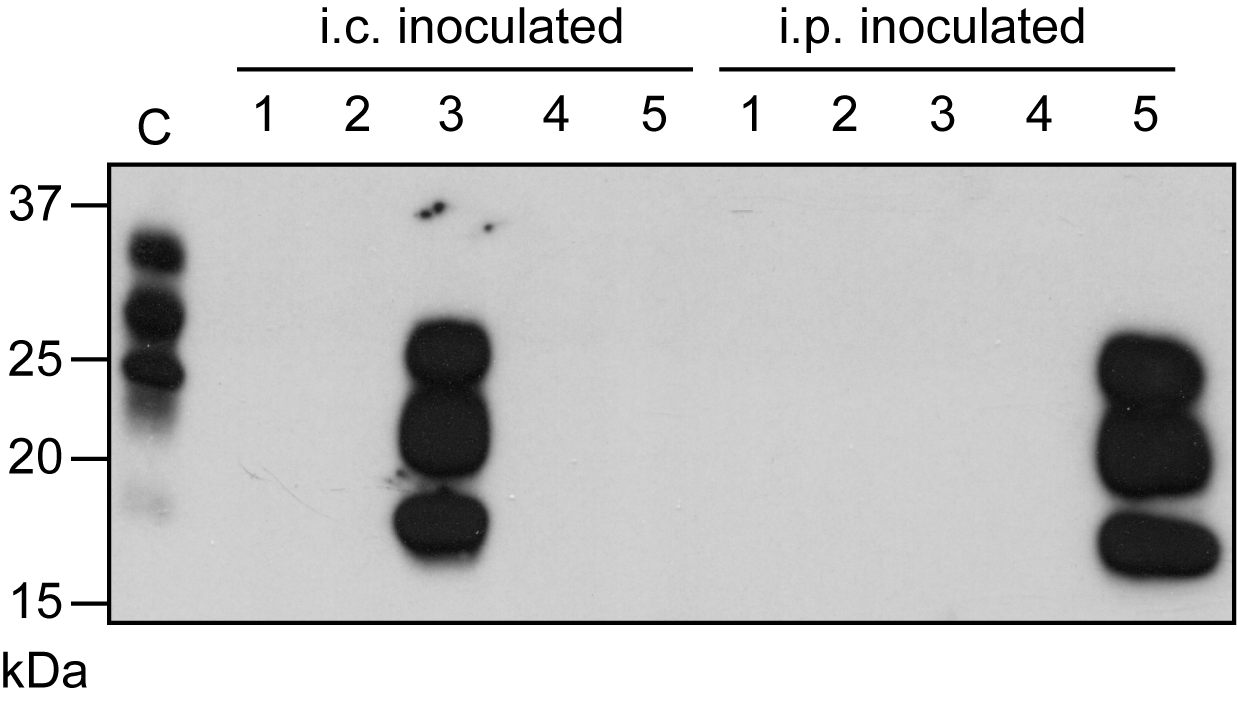

Supplement: S5 Fig — Brain homogenates prepared from wild-type CD-1 mice that received i.c. or i.p. inoculation of IP#152 mouse brain homogenate were subjected to PK digestion followed by immunoblot analysis. PrP was detected with POM1 anti-PrP antibody. The mice without any clinical signs were sacrificed after > 500 dpi. C, undigested mouse brain homogenate used as a control. (TIF) [file ppat.1004958.s005.tif]
