# Supplementary material for: Intraperitoneal Infection of Wild-Type Mice with Synthetically Generated Mammalian Prion
Source: PLoS Pathog. 2015 Jul 2;11(7):e1004958. doi: 10.1371/journal.ppat.1004958 (PMC4489884; doi:10.1371/journal.ppat.1004958)
Supplement: S1 Method — (DOCX) [file ppat.1004958.s006.docx]

**S1 Method**

Un-tagged recPrP purification ⎯ Transformed cells were cultured in 1 liter LB medium till OD_600_ reached 0.5-0.6. Afterward, IPTG was added to reach final concentration of 1 mM and the culture was continued for 5 hours. Induced *E. Coli* cells were harvested (5000g, 20 minute, 4ºC) and stored at -80ºC. For purification, *E. Coli* cells were resuspended in 75 mL of buffer A (10 mM Tris–HCl, 100 mM NaPO_4_, pH 8.0) and lysed through 4 rounds of 2-minute sonication on ice (Branson Sonifier 450, output = 6 and duty cycle = 80%, 10-minute incubation on ice between each round). Inclusion bodies were collected by centrifugation (15,000g, 30 minute, 4ºC), resuspended in 75 mL of buffer B (6 M GuHCl, 10 mM Tris–HCl, 100 mM NaPO_4_, 10mM BME, pH 8.0), and sonicated on ice till completely solubilized (4 rounds of 4-minute sonication, output = 6, duty cycle = 80%, Branson Sonifier 450, 10 minute break between each round). After centrifugation (15,000g, 30 minute, 4ºC), the supernatant was mixed with 25 mL of Ni–NTA Superflow resin (pre-equilibrated with buffer B) and stirred for 30 minutes at room temperature. The resin was then packed into a glass chromatography column and washed with 120 mL of buffer B. Afterward, a 200 mL gradient of buffer B to buffer A was applied to the column (2 mL / minute), which was followed by a wash with 50 mL buffer A. The recPrP was eluted with elution buffers with increasing imidazole concentrations, which contains 10 mM Tris–HCl, 100 mM NaPO_4_, pH 8.0 plus 50, 100, 150, 250 or 500 mM imidazole. The eluted fractions with OD_280_ ≥ 0.1 in each elution buffer (usually in elution buffer contains 50, 100, or 150 mM imidazole) were combined, added to Dialysis Tubing (10,000 MWCO), dialyzed for 1.5 hour against buffer E (10 mM NaPO_4_, pH 5.8, changing buffer every 30 minute) and another 1.5 hour against ddH_2_O (changing ddH_2_O every 30 minute). Dialyzed recPrP in ddH_2_O was kept at -80ºC.
