# Supplementary material for: Intraperitoneal Infection of Wild-Type Mice with Synthetically Generated Mammalian Prion
Source: PLoS Pathog. 2015 Jul 2;11(7):e1004958. doi: 10.1371/journal.ppat.1004958 (PMC4489884; doi:10.1371/journal.ppat.1004958)
Supplement: S1 Table — (DOC) [file ppat.1004958.s007.doc]

**S1 Table** Controls for Experiment 1

| **Inoculum** | **Component** | **Processing** | **Preparation for inoculation** | **Diseased / Inoculated** |
| --- | --- | --- | --- | --- |
| Negative control **1** | Buffer + POPG + RNA * | sPMCA | Pelleting through a sucrose cushion and washing twice with PBS | 0/5 |
| Negative control **2** | Buffer + POPG + RNA + recPrP * | Incubated at 37°C without sonication | Pelleting through a sucrose cushion and washing twice with PBS | 0/5 |
| Negative control **3** | POPG + RNA+ recPrP ** | No processing | No preparation | 0/5 |
| **rec-Prion** | Buffer + POPG +RNA+ recPrP + rec-Prion seed | sPMCA | Pelleting through a sucrose cushion and washing twice with PBS | 10/10 |

* The amount of each component equaled that in the rec-Prion propagation reaction.

** The amount of each component equaled that in the final pool of the rec-Prion inoculum.
